# Supplementary material for: Evaluating the use of social contact data to produce age-specific short-term forecasts of SARS-CoV-2 incidence in England
Source: PLoS Comput Biol. 2023 Sep 12;19(9):e1011453. doi: 10.1371/journal.pcbi.1011453 (PMC10516435; doi:10.1371/journal.pcbi.1011453)
Supplement: Attachment — Submitted filename: review_PLoSCompBio_01784.pdf [file pcbi.1011453.s004.pdf]

Referee report for "Evaluating the use of social contact data to produce age-specific forecasts of SARS-CoV-2 incidence" by Munday et al.

Luiz Max de Carvalho

January 19, 2023

Abstract

In this paper, Munday and colleagues investigate the forecasting performance of models for COVID-19 which incorporate detailed contact information. They use data from the CoMix survey which is a dynamic survey as opposed to static contact matrices that are usually employed in this kind of model. They find that using the contact information leads to better short-term forecasts in certain cases (certain targets in certain periods) but there is a lot of heterogeneity in performance. Overall, including contact information might be useful for longer horizons (2 to 4) weeks.

Overall impression

The paper is quite well-written, the goals are clear and the results are well-presented, at least in the text. I found most of the figures more confusing than helpful. I think the main message is: contact info helps sometimes, and that is a solid finding; One thing missing from the discussion is whether including contact information is worth it in analytical terms: the authors already point out that their assessment does not transfer well to the surveillance setting because they use "complete" data as observed after the fact and not the actual data decision-makers would have had access to at the time. And then it is not clear how *much better* each model was than the other in the situations where the CoMix-informed model "won". Is it worth the hassle? I mean, does it matter that a model had a rCRPS of, say, 0.9? Is that significantly better than the model it is being compared to? In other words, it is not clear to me if the differences in ranking between the models (i.e. who's got the lowest CRPS) translate to **meaningful** differences in terms of decision-making? I would appreciate if the authors could broach these questions in their already quite lengthy Discussion section.

I have added a marked up PDF with some comments.
Disclaimer:

Since the identity of the authors is known to me, I will be signing this review. Note also that not all comments are criticisms, if would believe such a thing is possible in a referee report.

Major comments

M1 Line 24: I quite like the idea of using and emphasising proper scoring rules;

M2 Lines 131 and 133: What fourth model? I'm confused.

M3 Line 160: What's meant by "infection prevalence" here? I understand "antibody prevalence" is a measure of cumulative exposure (bar loss of immunity and test imperfections), but I don't understand what "infection prevalence" means here.

M4 Figure 1: What information does the third panel at the bottom bring? I'd stick with incidence. Also, what exactly is the scale here? Cases per 100K?

M5 I could not find the meaning/definition of $A_a(t)$;

M6 Line 221: Technical point: symplectic integrator divergences have nothing to do with HMC convergence; they are however a good diagnostic for integration problems and I'm glad the authors paid attention to this. Also, "chain mixing" is never defined or explained. We all have an intuitive understanding of what this means, but it pays to be precise. Did the authors look at effective sample size?

M7 Line 243: Notation is a bit confusing here (too many subscripts); how exactly is $C_{\sigma,ab}$ estimated? Does it have a prior? Is it a standard normal one?

M8 I absolutely abhor the use of the expression "uninformative priors". There is no such thing as uninformative priors! I would sim-ply say something along the lines of "The priors we employed are given in Table 1". Should the authors want to justify their prior choices, I suggest they do so by simulating from these priors and showing that the resulting predictives encompass a wide range of plausible data configurations.

M9 I think it would be nice if the authors could add a 'reference' column to Table 1, where references to the data/rationale employed in the elicitation could be more readily accessed.

M10 Line 302: I like the idea of using a relative CRPS.

M11 Figure 2 is very low res. Hard to read on a tablet, for instance.

M12 The left panel Figure 3 is packed with information, but unfortunately it is really hard to parse all of the information. I suggest the authors re-work their figure to show one thing and one thing only, and show it well. Also, what's with the squiggly lines in the right panel? Why is there a 'sigmoid' between each point?

M13 Figure 6 follows in the footsteps of its predecessors in being absolutely inscrutable to me; what exactly do the authors want to show here?

M14 Line 553: I think the point of heterogeneity is a good one, and is not emphasised enough in the literature. A little citation in that direction would make this sentence even better, though.

Minor comments

m1 Line 10: it's obvious forecasts can provide predictions. I assume the authors meant something like "insights".

m2 Line 12: I assume 'They' refers to 'models', in which case the sentence does not make much sense, since models do not infer anything. We do.

m3 There are many instances of references with no space between the word immediately preceding the reference and the reference itself.

m4 Many display equations are also missing proper punctuation.

m5 Lines 71 to 75: confusing paragraph, with too many commas in the wrong place.

m6 Line 74: I think the problem is more than the dimensionality here. Is it not an identifiability problem also?

m7 Line 238: repeat of line 231.

m8 Line 291: I think it is easier to say "(henceforth called 'coverage')" to indicate what exactly is being referred to.

m9 Line 445: is 'present' the best word here?

m10 Line 522: one can acquire immunity without acquiring antibodies; do the authors mean antibodies when they mean overall immunity?

m11 Forgive my ignorance, but what is 'date of specimen'? A cursory internet search did not turn up a definition.
