# Supplementary material for: Evaluating the use of social contact data to produce age-specific short-term forecasts of SARS-CoV-2 incidence in England
Source: PLoS Comput Biol. 2023 Sep 12;19(9):e1011453. doi: 10.1371/journal.pcbi.1011453 (PMC10516435; doi:10.1371/journal.pcbi.1011453)
Supplement: Attachment — Submitted filename: Response to reviewers .pdf [file pcbi.1011453.s005.pdf]

Response to reviewers - Evaluating the use of social contact data to produce age-specific short-term forecasts of SARS-CoV-2 incidence in England.

Thank you for your comments, I have included our responses below in green and relevant text relating to the major comments in blue.

Reviewer 1:

Major comments:
My main concern is that the manuscript is very descriptive (e.g. the authors show us which method seems to provide better forecasts during which time periods), but the manuscript lacks insights and a discussion why this could be the case.

1) This could be addressed in describing a little more what the major differences are between the Comix and Polymod matrices. Did the authors attempt to use the "physical only contact" matrices from Polymod? Would they be more similar to the Comix matrices over long time periods ?

We thank you for your comment, we agree that the details of the difference between POLYMOD and CoMix contact data is missing. We have added a section addressing this in the methods. We also refer the reader to a previous paper by Gimma et. Al. for more in depth analyses of the two data sets. Line 136

To evaluate the benefit of regularly collected contact data over interactions informed by previously collected contact data, we implemented a second instance of the same model using data collected in the POLYMOD survey. This survey conducted in 2008 was designed to measure rates of contact in the population for parameterisation of mathematical models of infectious transmission. The POLYMOD data represent contact during a non-pandemic period and in our study represented an interaction assumption that may be made if the regularly collected CoMix data were not available. Gimma et. al. [19] compared CoMix survey rounds and POLYMOD data in detail, showing that the mean contact rates varied substantially in all age groups between CoMix and POLYMOD, but also between the rounds of CoMix. In particular, the relative rate of contact in children varied between rounds. For example, compared to POLYMOD, there was a much greater reduction in childrens' contacts than adults' during periods that schools were closed, however as schools re-opened the childrens' contacts almost returned to parity with POLYMOD, whereas adults' contacts remained reduced. Hence the CoMix data added information on both time-varying mean contacts and variation in the interaction between age-groups.

2) I was missing also a more indepth analysis of performance as a function whether the outcome statistic is "increasing", "stable" or "decreasing". This is

only alluded to in the final paragraph of the discussion. I think it would be useful to integrate this more into the analysis. This might make the paper more interesting for persons wanting to use forecasting for different purposes and different diseases.

We are grateful for your insightful point. We are generally opposed to scoring forecasts based on their performance during particular characteristic phases of an epidemic. This is because by doing so there is a danger that you will select for the model with the most convenient error for that particular characteristic. e.g. for the decreasing phase it is likely that a model which consistently under predicts incidence will perform best. However, to address your point, we have added descriptions of the epidemic trajectory during each phase, to make this effect clearer in our interpretation of the forecast evaluation (See table 2). We have also addressed this issue in the discussion appending the relevant paragraph with the following text (LINE 550):

"Christmas and Lockdown 3 is also the only period of sustained decline in infections, this may indicate that the CoMix model performs best during the declining phase of an epidemic. However, this isn't supported by the forecast performance in the lockdown 3 easing period, where the performance of the CoMix informed model was best when infections began to increase again."

3) While the statistical methodology seems appropriately chosen, it is not easy to gauge the "significance" of the observed differences of CRPS between the models. Could the observed differences have arisen by chance (e.g. there are clear sample size differences for the different pandemic periods)? There are obviously differences in degrees of freedom when fitting data - as Comix matrices vary over time and POLYMOD stay the same. Isn't there a risk of overfitting, which is the most parsimonious approach ? What are the criteria to determine whether a method is better, overall, in light of these differences?

Thank you for your comment. Unfortunately, since CRPS is not a statistical test, so calculating a significance is not feasible. Rather they reflect precisely how the performance of the different forecasts compared for the specific example we show. To aid the reader's understanding, we have included the following paragraph in the supplementary information:

"Proper scoring rules are not statistical tests of general forecast performance but evaluate the performance of each model in a particular forecasting task. For this reason there is no associated uncertainty or "significance" measure to present."

We are not concerned with overfitting as a result of the increased information from CoMix because although we add more data, the number of parameters in the model has not increased. Therefore, the addition of this information does not allow additional flexibility within the model but rather constrains the model differently.

4) In addition to Fig. 6, it would be useful to also provide graphs of estimated average infectiousness and susceptibility over time (possibly as supplementary information) to support statements like in line 520.

Thank you for raising this. Figure 6 does show progression through time but hue however it is clear to us that the plot was not intuitive to interpret so we have now reworked figure 6 to show infectiousness and susceptibility by forecast date for younger and older adults separately.

5) I was a missing a discussion of what the findings mean for public health and for pandemic preparedness. Are such forecasts useful and what do the authors suggest should be planned ahead for the next pandemic. Will we need contact surveys and infection prevalence surveys?

Thank you kindly for raising this to our attention. We have added the following sentence to the discussion to highlight our recommendations for priorities of policy decision makers (Line 684):

"In summary, our analysis broadly supports the collection of both infection prevalence study data and contact data and their use in short-term epidemic forecasts, but also highlights the limitations of this data. We acknowledge that these studies are resource intensive and we therefore advocate for further in-depth evaluation of contact survey design to ensure they capture changes in behaviour that closely reflects changes in transmission."

Minor comments:

- Wrong years in abstract line 23 p.1 (october 2021 and November 2022 ?).
Thank you for your thoughtful comment, these dates are actually correct. We studied data prior to the emergence of omicron, which occurred in December 2021.

- Figure 1: the right hand side legends indicating the colour for each age group is hard to read. Actually it is also a bit superfluous as the age groups are indicated clearly on top of each graph. I suggest to remove the rhs legend.
Thank you for your helpful comment, we agree and have removed the legends in figure 1.

- From 2021 onwards, seroprevalence is also influenced by vaccination, so it would be necessary to add a supplementary file with cumulative vaccination coverage per age group to Figure 1.
Thank you for your helpful suggestion. We have now added a figure showing the number of vaccinations on each day in the UK for each of the ONS age groups, to the supplementary information - Figure S1.

Reviewer 2:

Major Comments:

M2 Lines 131 and 133: What fourth model? I'm confused.

Thank you for pointing out this error. The first sentence refers to the third model and the second to the fourth model we describe. We have adjusted the text.

M3 Line 160: What's meant by "infection prevalence" here? I understand "antibody prevalence" is a measure of cumulative exposure (bar loss of immunity and test imperfections), but I don't understand what "infection prevalence" means here.

Thank you for raising this to our attention. We have now clarified the definition of infection prevalence in the manuscript (Line 187):

"Here infection prevalence is defined as PCR test positivity prevalence under the protocol of the CIS."

M4 Figure 1: What information does the third panel at the bottom bring? I'd stick with incidence. Also, what exactly is the scale here? Cases per 100K?

We are grateful that you have raised this confusing point. The third panel actually shows the reported cases from the UK COVID-19 Dashboard - which is a separate data source and is the data we refer to when we apply the model to 'case data' we have clarified this in the figure caption.

"Fig. 1. Estimated incidence of A) infection, B) antibody prevalence from the ONS Community Infection Survey (CIS) and C) case reports from the UKHSA COVID-19 Dashboard, each shown by age group."

M5 I could not find the meaning/definition of Aa(t);

Thank you for bringing this omission to our attention. We have now added the following definition to the text (Line 245):

"and Aa(t) is the antibody prevalence in age-group a at time t."

M6 Line 221: Technical point: symplectic integrator divergences have nothing to do with HMC convergence; they are however a good diagnostic for integration problems and I'm glad the authors paid attention to this. Also, "chain mixing" is never defined or

explained. We all have an intuitive understanding of what this means, but it pays to be precise. Did the authors look at effective sample size?

Thank you for raising this point. We have now amended the text and have separated the reference to divergent transitions from the reference to convergence metrics.

M7 Line 243: Notation is a bit confusing here (too many subscripts); how exactly is $C\sigma,ab$ estimated? Does it have a prior? Is it a standard normal one?

Thankyou for raising this. This is the measured standard deviation of contacts between age-groups a and b. We have clarified this in the text. (Line 287)

"and $C\sigma,ab$ is the observed estimated standard deviation of the measured contact rate and σcm is the uncertainty in the posterior contacts rates."

M8 I absolutely abhor the use of the expression “uninformative priors”. There is no such thing as uninformative priors! I would sim-ply say something along the lines of “The priors we employed are given in Table 1”

Thank you for your suggestion. We have updated the manuscript with your suggested text.

M9 I think it would be nice if the authors could add a ’reference’ column to Table 1, where references to the data/rationale employed in the elicitation could be more readily accessed.

Thankyou for your suggestion, we have now added a reference column to table 1.

M10 Line 302: I like the idea of using a relative CRPS.

Thank you for your comment, we like it too!

M11 Figure 2 is very low res. Hard to read on a tablet, for instance.

Thank you for your comment, we apologise for the resolution of the image in our manuscript. We have replaced it with a higher resolution image. We note that in the full submission all figures will be submitted as vector image files.

M12 The left panel Figure 3 is packed with information, but unfortunately it is really hard to parse all of the information. I suggest the authors re-work their figure to show one thing and one thing only, and show it well. Also, what’s with the squiggly lines in the right panel? Why is there a ‘sigmoid’ between each point?

Thank you for raising this. In light of your comment we have revised figure 3, splitting the axes in panel A into two. So now we show overall rCRPS and Bias on separate axes, which makes it easier to interpret. The sigmoids between the points are commonly known as a ‘bump plot’ (implemented with *ggbump*); they are becoming a popular way to show association between points without suggesting interpolation. We like them so we have kept them in for now.

M13 Figure 6 follows in the footsteps of its predecessors in being absolutely inscrutable to me; what exactly do the authors want to show here?

Thank you for raising this, we have reworked figure 6 which now shows infectiousness and susceptibility by forecast date for younger and older adults separately.

M14 Line 553: I think the point of heterogeneity is a good one, and is not emphasised enough in the literature. A little citation in that direction would make this sentence even better, though.

The authors agree that this is an important point and we are glad that we have raised it. We have not been able to find a study that approaches this question directly. We would be very happy if the reviewer could suggest one to us.

Minor comments:

m1 Line 10: it’s obvious forecasts can provide predictions. I assume the authors meant something like “insights”.
Thank you for raising this point. We have adapted this sentence as follows (Line 10):
"Mathematical and statistical models can be used to make predictions of how epidemics may progress in the near future, and form a central part of outbreak mitigation and control."

m2 Line 12: I assume ’They’ refers to ’models’, in which case the sentence does not make much sense, since models do not infer anything. We do.
Thank you for highlighting this. We have altered the text (Line 12) :
"Renewal equation based models allow inference of epidemiological parameters from historical data and forecast future epidemic dynamics without requiring complex mechanistic assumptions."

m3 There are many instances of references with no space between the word immediately preceding the reference and the reference itself.
Thank you for highlighting this stylistic point. We have reviewed the manuscript and corrected these errors.

m5 Lines 71 to 75: confusing paragraph, with too many commas in the wrong place.

Thankyou for highlighting this. We have adjusted the paragraph as follows (Line 72):
"Throughout the pandemic there has been a great deal of variance in prevalence of infection between age groups. Although this further motivates the requirement for age-specific forecasts, especially to better understand the risk to particularly vulnerable groups, this heterogeneity in prevalence also suggests that transmission rates between age groups may need to be captured to effectively make such forecasts. However, the high dimensionality of this problem means that the full age interaction matrix is not identifiable from epidemiological data alone [17]."

m6 Line 74: I think the problem is more than the dimensionality here. Is it not an identifiability problem also?
Thank you for raising this point. We have now reworked this paragraph. See previous response.

m7 Line 238: repeat of line 231.
Thank you for raising this. Yes they are very similar sentences however one refers to the model of case reports and the other refers to the model of infection estimates and so they are both necessary in the manuscript.

m8 Line 291: I think it is easier to say "(henceforth called 'coverage')" to indicate what exactly is being referred to.
Thank you for this valuable contribution. I have changed the text as suggested.

m9 Line 445: is 'present' the best word here?
Thankyou for this comment, we have reworded the text (Line 498):
"The baseline models are not presented as they do not include confidence ranges"

m10 Line 522: one can acquire immunity without acquiring antibodies; do the authors mean antibodies when they mean overall immunity?
Thank you for raising this, we have changed the text as suggested referring to acquired immunity as opposed to antibodies.

m11 Forgive my ignorance, but what is 'date of specimen'? A cursory internet search did not turn up a definition.
Thank you for raising this to our attention. I have recently been made aware that 'date of specimen' Is quite a UK centric term. It refers to the date at which the swab was taken. To make this clearer and to avoid confusion with the date that the PCR was conducted I have changed the text to (Line 648):
'the date at which individuals are swabbed'

Reviewer 3:

Major Comments:

Title: I think the title should be updated by replacing "forecasts" to "short-term forecasts", and by adding the country name. Since this approach seems to be very specific to UK data.
Thank you for your suggestion. We have changed the title to reflect this.

Data sources: For COVID-19 cases, the data come from the UK COVID-19 dashboard, it is the usual number of COVID cases data, are cases reported on that week or cases whose onset symptoms occur on that week? What was the date associated with a case? The weekly infection incidence is an estimate and it is a function of SARS-CoV-2 prevalence and antibody prevalence collected as part of an infection survey, and since it is a survey there is must be some uncertainty on these estimates. And such uncertainty is ignored in the proposed model.

Thank you for raising these points. We are aware now that we have not communicated the model sufficiently clearly. We have added a line to clarify how the case data is dated. You are right that the infection incidence is an estimated quantity and therefore has uncertainty associated with it. We account for this uncertainty directly in the model by making the infection timeseries a vector parameter in the stan model, the prior of this vector is then defined by the mean and standard deviation of the infection time series that we have estimated using inc2prev. We Have added a sentence in the data section to reflect this to avoid confusion for the reader from the outset (Line 201):

"We accounted for the uncertainty in the estimated antibody prevalence and infection incidence by including the time-series' as parameters in the model, with the prior at each day informed by the estimated distributions above (see the Transmission Model section of this paper for more details."
No contact data: C(t) ? This one I did not understood how the contact matrix was estimated.

Thank you for raising this point. We agree that we have not made this part of the model sufficiently clear in the text. To answer your question: The no-contact data based interaction matrix is estimated by the model using a naïve prior gamma(2,2) for each element. We anticipate that this interaction matrix will not be an accurate estimate due to the previously discussed identifiability problems with this inference, however, since we do not intend to use this model for inference of contact rates, it may be that the over-fit interaction matrix performs better in a forecasting context. We have clarified this section of in the text (Line 152):

"In the third model the interaction between age groups was not informed by contact data at all, but estimated entirely from historical epidemiological data. We do not anticipate the inferred contact matrix to necessarily reflect the true contact rates due to the aforementioned challenges with identifiability. Rather, we wish to test if over-fit contact matrix from this model performs better than the model based on the collected data."

The manuscript notation is not simple to follow, and there are some typos. There is no difference among, scalars, vectors and matrices in the equations, so it is not clear that I(t) in equation (1) is a vector where each element contains the estimated incidence for each age-group at time t. In the text, it is clear that N(t) and C(t) are matrices but it is not clear in equations 1, 3, 6,and 9.

Thank you for raising this. We have now changed the notation. To clarify the use of vectors and matrices we employ bold font for matrices and annotate vectors with an arrow.

In equation (3), it is not clear what diag(s) or diag(i) means. I assume they are diagnoal matrices with vectors s and i in that diagonal. Again, it is not a clear notation.

Thank you for taking the time to check this. We are of the understanding that diag(v) is commonly used to describe a diagonal matrix with diagonal described as vector v. We use the same notation as previous literature that represents use of augmented next generation matrices in the same way. Franco N, Coletti P, Willem L, Angeli L, Lajot A, Abrams S, et al. Inferring age-specific differences in susceptibility to and infectiousness upon SARS-CoV-2 infection based on Belgian social contact data. PLoS Comput Biol. 2022;18: e1009965.

Equation (4), in $s_{ab,a}$ what does b mean?

Thank you for drawing our attention to this poorly defined variable. We have clarified the description in the text (Line 239):

"The first (sab,a) is the drawn from age-specific acquired immunity to infection in age group a, thiswhich is informed by weekly age-specific antibody prevalence estimates from inc2prev."

Equation (5), function $A_{a}(t)$ is not defined. In table 1, it is called antibodies. I assume it is the antibody prevalence for age-group a at time t, and it follows a [0,1] truncated Gaussian distribution with undefined mean and variance. (Table 1)
Thank you for bringing this omission to our attention. We have now added the following definition to the text (Line 245):

"and Aa(t) is the antibody prevalence in age-group a at time t."

We have also clarified how the antibody prevalence is estimated in our model (Line 251):

"To account for uncertainty in estimates of antibody prevalence, Aa(t) was also estimated in our model, the prior was based on the mean and standard deviation of the previously estimated antibody prevalence using inc2prev (Aμ,a, Aσ,a). "

Priors in table 1. In page 11, line 250, the authors claim to use uninformative priors for C_{aa}, (CV_I, CV_C and \sigma_{cm}) and \Phi. They do not seem to be uninformative. I am not sure if a large prior sensitivity analysis would be required here, but it would be interesting to explore for a couple of those parameter a set of different priors with smaller and greater variances in at least one of the proposed models to check how robust is the inference. Antibody protection (Phi) and CV_I or CV_c.
Thank you for raising this important point. We have amended the text to remove the reference to uninformative priors.

In equation 8, it seems to be a circular problem by using the I_\mu(t) random vector as a function of its own standard deviation. Perhaps in equation (6) the random vector is I(t) with mean I_\mu(t) which should be equation (1).

Thank you for bringing our attention to this. The uncertainty is modelled as COV in order to scale it with the overall infection numbers - which vary substantially. Here we use the mean of the estimated infections input into our model to scale the uncertainty - rather than the estimates from our model themselves. This avoids the circular issue you raise. We have clarified this in the text (Line 272).

"Where Iμ,a(t) is the mean of the infections estimated by inc2prev in age group a. This ensures the uncertainty scales with the magnitude of the infection incidence estimates."

Equation 10, I believe it should be $\sigma_c(t)$ instead of $\sigma_I(t)$.

Thankyou for bringing this to our attention, we have corrected this in the text.

For model evaluation (page 18) the authors use two other "models" as baseline. They are not really models unless it is explicitly said that the probability distribution for those estimates is a zero variance variance distribution centred on the proposed estimates. Why not add a Gaussian noise to make it random, or perhaps use a usual statistical time series data-driven model to do k-step ahead forecast. Then I would suggest an ARIMA-like multivariate model or a Bayesian linear dynamical model as West and Harrison (1997, https://doi.org/10.1007/b98971). The advantage is that a baseline statistical model would be better for a comparison. For instance, I wonder if an s_{max} order autoregressive model would be too different than an NGM transmission model with no interaction. Also the uncertainty for the k-step ahead forecasts would also be dealt with since it is an statistical model after all.

Thank you for engaging with us on this. We agree that there are many model specifications that could be compared in our analysis. We have thought hard about

which to include in this paper. Firstly regarding the baseline models. The purpose of these models is to provide an indication of each model performance against a naive assumption which might closely mirror that which is perceived by a public health professional making predictions about the epidemic. We therefore chose the two most clearly defined simple assumptions which might be applied. No change from the forecast date and exponential growth. We chose not to include any uncertainty in this estimate as it is not clear how this should be calculated and we believe that a single point estimate is reflective of 'drawing a line through the data', which is a common instinct for rule of thumb predictions. Secondly, we want to address the use of a traditional forecasting model. We thank the reviewer for this suggestion. We considered this at length. The four models we chose for this analysis all reflect the same NGM structure with varying data to inform them. We wish to concentrate on the use of contact data to inform transmission between age-groups. The strength of our current approach is clarity - it allows us to evaluate the role of particular model components. We see the fourth model as the closest model to your suggestion of a time series model, with each age-specific time-series informed only by its own history. Since we already compare 6 forecasts (4 NGM models and 2 baseline models), and due to the similarity between the time-series model and the fourth NGM model, we feel strongly that introducing additional models unnecessarily complicates the analysis. We appreciate that this is a clear analysis decision and we have now addressed it in the discussion (Line 656):

" In addition, there are many alternative model specifications that could have been incorporated into our analysis. In the interest of clarity we chose to include only NGM based models, emphasising our evaluation of the use of contact data and not model specification. "

Still on model evaluation (page 18), the usual continuous ranked probability score (CRPS) is a scoring rule for an univariate quantity, so for a given age-group and a k-step ahead forecast the CRPS would be calculated using the posterior predictive distribution of incidence I_a(T+k) where a is the age group, T is index for the last observed week and k are the number weeks ahead to forecast (analogously it works for cases c_a(t)). In this case, the CRPS is clear, they are presented in Figure 3B for instance. I am not sure how the CRPS was calculated for the multivariate version, which are the main likelihoods in the manuscript (equations 6 and 9). Is it a multivariate CRPS or is it a CRPS calculated over a function of the vector leading to a scalar, for example I^*_(T+k) = \sum_a I_a(T+k) which would be the total incidences forecasted for week T+k. This is important to clarify because I am not sure what I am looking in Figures 2C, 3A and 4. Also in Figures 2,3 and 4, even though the y-label says CRPS it should be rCRPS.

Thank you for raising this with us. You are right that we failed to define the combined CRPS in this paper. To be clear, it is calculated as the average CPRS across every forecast date and age group. This is a well established approach for scoring forecasts.

This essentially extends the existing definition from averageing the CRPS on a temporal bases to averaging across age-groups as well. We have clarified this in the text (Line 331):

" To summarise the performance of the entire model, we calculated the overall CRPS as the mean CRPS across all age-groups "

Forecast calibration (page 22). All models underestimate the uncertainty (Figure 5A), and the proposed model behaves in a counter intuitive fashion. I would expect that the credible intervals would increase as we increase the horizon, and therefore we would observe more points inside the intervals until we get an intervals that are so large that any point would in it the proportion of points inside the interval would converge to 100%. That is not what is happening in the proposed models, of course data could lead to strange behaviours this is one of the reasons I would like to see one or two usual statistical time series model as baseline models.

Thankyou for raising this point. We understand your concern. These plots show the proportion of points that fall within the 50% and 90% central ranges. As the credible intervals increase, the model becomes more uncertain about the true values. On this plot a 'perfectly' calibrated model will produce a horizontal line with the intercept at the value of the central range (shown in grey on each plot). Therefore a model can have increasing uncertainty with decreasing coverage in the event that the uncertainty is not increasing sufficiently to capture the true values. This is what is happening in our case.

In Figure 6, I am not sure what is meant by infectiousness and susceptibility here. In page 24, the authors extract these quantities from the "model fits". What does model fits mean? Is this a predictive posterior quantity? Which one? Or is it a function of some estimated parameters?

Thank you for bringing the poor wording of this section to our attention. We have now clarified the text (Line 510):

"We extracted posterior estimates of time varying age specific infectiousness (i) and susceptibility (s) to assess the biological plausibility of these parameters (Figure 6).. "
