# Supplementary material for: Evaluating the use of social contact data to produce age-specific short-term forecasts of SARS-CoV-2 incidence in England
Source: PLoS Comput Biol. 2023 Sep 12;19(9):e1011453. doi: 10.1371/journal.pcbi.1011453 (PMC10516435; doi:10.1371/journal.pcbi.1011453)
Supplement: Attachment — Submitted filename: Response to reviewers - round 2.docx [file pcbi.1011453.s006.docx]

Dear Editors,

Thank you for taking the time to consider our manuscript. We are thrilled to hear that it is likely to be accepted. Please find our response to the reviewers and to the additional editorial points below. Do let us know if there are any other adjustments to be made.

Sincerely

James Munday (on behalf of the authors)

Response to reviewers – round 2

Reviewer #2: I would like to warmly compliment the authors on their thorough revision work. I'm pleased with the changes made to the paper and genuinely think it'll be a much better piece because of them. Unfortunately, I do not have a reference to recommend on the issue of heterogeneity in antibody levels across age-strata, so I suppose we will have to drop the issue for now.

Thank you for your thoughtful and constructive comments. We agree that the manuscript is improved as a result.

Reviewer #3: The authors have answered all of my questions. The new notation has improved a lot my understanding of the proposed models.

In general, the CoMix model was still the best model for infection incidence from prevalence survey data. However for cases, adding age interactions and contact data did not improve performance according to CRSP. I understand the CRPS is an usual measure to evaluate model performance. The authors correctly pointed that out. But in my experience, a probabilistic model that represents/emulates the complexity of real data would lead to a reduction of predictive uncertainty (captured for instance in a reduction of the range of the prediction intervals), which in my humble opinion would be a better improvement. I am not sure if the CRPS would capture such improvement.

I am not suggesting to try a different predictive measure, that would imply on a chance of the baseline model. That is why I suggested to change it to a simpler probabilistic model like an ARIMA or a state-space model. Again I understand the author argument of using far more basic (near deterministic) models and I am OK with that argument. So I am happy with the manuscript the way it is now.

Thank you for your helpful comments and feedback. We are glad that we were able to come to an agreement on the above points. We very much appreciate the time and effort you have committed to improve this manuscript.

Typo:

Line 226. After equation 3. veci probably should be \vec{i}

Thanks for raising this with us, we have now corrected this in the revised manuscript.

Response to editorial instructions

Figure Files:

While revising your submission, please upload your figure files to the Preflight Analysis and Conversion Engine (PACE) digital diagnostic tool, <https://pacev2.apexcovantage.com>. PACE helps ensure that figures meet PLOS requirements. To use PACE, you must first register as a user. Then, login and navigate to the UPLOAD tab, where you will find detailed instructions on how to use the tool. If you encounter any issues or have any questions when using PACE, please email us at [figures@plos.org](mailto:figures@plos.org).

Thank you for drawing our attention to this resource. We have now used the PACE tool to bring the figures in line with editorial policy. Please find the new figure files submitted.

Data Requirements:

Please note that, as a condition of publication, PLOS' data policy requires that you make available all data used to draw the conclusions outlined in your manuscript. Data must be deposited in an appropriate repository, included within the body of the manuscript, or uploaded as supporting information. This includes all numerical values that were used to generate graphs, histograms etc.. For an example in PLOS Biology see here: <http://www.plosbiology.org/article/info%3Adoi%2F10.1371%2Fjournal.pbio.1001908#s5>.

Thank you for highlighting this requirement. We now include all data relevant for plotting figures as an xlsx file in the supplementary material (S2 Data) we have also included a caption in the Supplementary materials section of the manuscript

Reproducibility:

To enhance the reproducibility of your results, we recommend that you deposit your laboratory protocols in [protocols.io](http://protocols.io), where a protocol can be assigned its own identifier (DOI) such that it can be cited independently in the future. Additionally, PLOS ONE offers an option to publish peer-reviewed clinical study protocols. Read more information on sharing protocols at <https://plos.org/protocols?utm_medium=editorial-email&utm_source=authorletters&utm_campaign=protocols>

Thank you for raising this. We do not have a lab protocol since this is an insilico study. Instead we have made the code publicly available, which allows the reader to re-run the entire analysis if they wish.

References:

Review your reference list to ensure that it is complete and correct. If you have cited papers that have been retracted, please include the rationale for doing so in the manuscript text, or remove these references and replace them with relevant current references. Any changes to the reference list should be mentioned in the rebuttal letter that accompanies your revised manuscript.

Thank you for raising this. We have checked the references and believe all are present and correct.

Additional modifications:

We have also corrected a few typos that we have found, most notably figure 1 which had a mislabeled panel. We have also added a new affiliation for James Munday and changed the correspondence address as the LSHTM correspondence email address is no longer active.
